# Effect of 5-azacytidine (5-aza) on UCP2 expression in human liver and colon cancer cells

**DOI:** 10.7150/ijms.56564

**Published:** 2021-03-19

**Authors:** Dae-Yeon Kim, Hee-Tae Cheong, Chang-Six Ra, Kazuhiro Kimura, Bae Dong Jung

**Affiliations:** 1 College of Veterinary Medicine & Institute of Veterinary Science, Kangwon National University, Chuncheon 24341, Korea.; 2 College of Animal Life Sciences, Kangwon National University, Chuncheon 24341, Korea.; 3 Faculty of Veterinary Medicine, Hokkaido University, Sapporo, Japan.

**Keywords:** 5-azacytidine, bisulfite sequencing, DNA methylation, methylation-specific PCR (MSP), UCP2, UCP2 promoter active.

## Abstract

The function of the uncoupling protein 2 (UCP2) is different for each cancer cell. However, the mechanism of expression is still unclear. DNA methylation affects protein expression and is one factor that transforms normal cells into cancer cells. In this study, the hepatocellular carcinoma Hep3B and HepG2 cells and colorectal cancer HT-29 cells were treated with 5-azacytidine (5-aza), a DNA demethylation agent, to observe the modification of UCP2 expression and the methylation degree in the UCP2 promoter region. Promoter basal activity and degree of UCP2 expression were measured in Hep3B, HepG2, and HT-29 cells. In addition, methylation-specific PCR (MSP) was performed to investigate the degree of methylation in the UCP2 promoter region. The methylation region in the UCP2 promoter was confirmed based on bisulfite sequencing. In Hep3B cells in which UCP2 mRNA was not transcribed, the promoter basal activity was significantly higher than in HT-29 or HepG2 cells in which UCP2 mRNA was transcribed. Treatment with 5-aza increased UCP2 expression in Hep3B and HT-29 cells; however, the expression in HepG2 cells was unchanged. The UCP2 promoter in Hep3B cells has numerous methylated regions compared with HT-29 and HepG2 cells. The results of the present study revealed that inhibition of UCP2 expression in Hep3B cells was due to methylation of the promoter region. Investigating the mechanism that induces UCP2 expression in cancer cells is important to understand the function of UCP2, which could aid in cancer treatment.

## Introduction

Since 2000, research has shown that 398,364 males and 165,972 females have developed liver cancer, and colon cancer has been reported in more than 1 million males and females worldwide 1, 2. Cancer-causing factors include genetic modifications such as methylation 3, 4. Methylation, an epigenetic variant, is found in the CpG dinucleotide gene promoter region and usually occurs in cytosine preceding guanine 5. DNA methylation is regulated by DNA methyltransferase (DNMT) 6, 7. Among the several types of DNMT, DNMT1, DNMT3a, and DNMT3b have been shown to regulate DNA methylation 8. These enzymes reduce the expression of proteins that inhibit cell proliferation. Consequently, cells start diving continuously and transform into cancer cells 4, 9, 10.

Uncoupling protein 2 (UCP2) is expressed in the inner membrane of the mitochondrion and transfers hydrogen ions from the inner membrane to the matrix 11. Studies have investigated the function of UCP2, but conflicting results have been reported. For cancer cells to grow, reactive oxygen species (ROS) production must be suppressed and glucose consumption should be increased 12. Alternatively, UCP2 can inhibit ROS production 13, 14. In one study, UCP2 inhibited the proliferation of cancer cells suppressing the activity of adenosine monophosphate-activated protein kinase/hypoxia inducible factor 15. In another study, UCP2 inhibited the proliferation of cancer cells by increasing ROS production 16. Therefore, UCP2 can play different roles depending on the cell type and situation. UCP2 expression also correlates with tumor modification 17. In breast cancer, UCP2 is significantly associated with tumor grade; increased UCP2 expression reduced the sensitivity of breast cancer cells to treatment 18, 19. In several studies, UCP2 was shown to play an important role in the resistance of pancreatic cancer to chemotherapy 20. These results indicate that regulation of UCP2 expression is strongly associated with the growth and treatment of cancer cells. However, the induction of UCP2 expression remains unclear.

The expression of UCP2 is suppressed in Hep3B, a human-derived liver cancer cell line, and is expressed in HepG2. In addition, UCP2 is also expressed in human-derived colon cancer cell line HT-29 11, 21. UCP2 expression can be influenced by various factors, and its expression is very closely related to its function. In this study, as part of studying UCP2 function, we first investigated the regulatory mechanisms for UCP2 gene expression. For this, Hep3B not expressing UCP2 and HepG2 and HT-29 cells expressing UCP2 were used. DNA demethylation drug 5-azacytidine (5-aza) was applied on these cells 22, 23 to measure the UCP2 promoter activity and UCP2 expression and the sequence of the methylated site was investigated through methylation specific PCR (MSP) and bisulfite sequencing.

## Materials and Methods

### Cell culture

The human HCC cell lines Hep3B (ATCC, Manassas, VA, USA; HB-8064) and HepG2 (ATCC, HB-8065) and human colon cancer cell line HT-29 (ATCC, HTB-38) were cultured on 100-mm culture plates (Falcon, Corning, NY, USA) in Dulbecco's modified Eagle's medium (DMEM; Sigma, Irvine, UK) containing 10% fetal calf serum (FCS; GE Healthcare Bio-Sciences AB, Uppsala, Sweden) and penicillin (100 μg/mL; GE Healthcare Life Sciences, Pasching, Austria) at 37°C in a humidified chamber with 5% CO_2_. 5-Aza (Sigma) was dissolved in deionized water (1 mM) and diluted to a concentration of 0, 5, or 10 μM in the culture fluid; cells were treated with different concentrations of 5-aza for 24, 48, or 72 h.

### Plasmid construct

The plasmids for the transient expression assay to examine basal promoter activity of the human UCP2 gene were constructed using the SEAP reporter system (TaKaRa, Tokyo, Japan) following the manufacturer's protocol. The following primers with appropriate restriction sites were used to amplify the promoter regions 21: sense primer sequence, 5′-GGTACCTCAAGATAACTGGTATGCCTTGT-3′, and antisense primer sequence, 5′-GAATTCTCATACTATGTGTCCGAGCCGCA-3′. PCR conditions were 40 cycles of 30 s at 94°C, 30 s at 60°C, and 3 min at 72°C, with a final extension of 10 min at 72°C. The PCR product size of UCP2 promoter was 2,960 bp. The PCR product was ligated into the *Kpn*I/*EcoR*I site of the polylinker region of the SEAP2 basic vector.

### Analysis of basal promoter activity of the human UCP2 gene

To examine basal promoter activity of the human UCP2 gene, transient expression assay of the UCP2 promoter SEAP construct was performed in Hep3B, HT-29, and HepG2 cell lines. The cells were cultured at a density of 1 × 10^5^ cells in 35-mm dishes and DMEM containing 10% FCS. After seeding, the dishes were washed extensively to remove non-adherent cells and the medium was replaced. On the second day, transfection was performed with FuGENE 6 transfection reagent (Boehringer Mannheim, Mannheim, Germany) according to the manufacturer's protocol. The plasmid (1 μg) consisting of the human UCP2 promoter region was fused to the SEAP basic vector and 1 μg of the SEAP control vector were co-transfected into each cells. After transfection, the medium was replaced and the cells cultured for an additional day. The supernatant was collected from each sample culture. The SEAP activity was measured with the chemiluminescent SEAP assay user manual (TaKaRa). Briefly, add 45 μl of 1 dilution buffer to each 15 μl sample and mix gently. The mixture was incubated at 65°C for 30 min, and then 60 μl assay buffer was added to each sample and again incubated for 5 min at room temperature (RT). After preparing 1.25 mM chemiluminescent substrate by diluting 1:20 with chemiluminescent enhancer, and then 60 μl of the diluted substrate was added to each sample, and incubated for 10 min at RT. The chemiluminescent signal was measured using a NanoDrop (Molecular Devices, LLC, Sunnyvale, CA, USA).

### Total RNA extraction and cDNA synthesis

Cells were homogenized with 1 mL of TRIzol Reagent (Ambion, Carlsbad, NM, USA), mixed with 0.3 mL of chloroform, and centrifuged at 12,000 rpm for 15 min at 4°C. The aqueous layer was transferred to a new tube and 0.6 mL of isopropanol was added. The tubes were then inverted several times and centrifuged at 12,000 rpm for 10 min at 4°C. The supernatant was removed and the RNA pellets washed with 70% alcohol. The RNA pellet was briefly air-dried and dissolved in diethyl dicarbonate-treated water. The total RNA concentration was measured using a NanoDrop (Molecular Devices). Complementary DNA (cDNA) was synthesized using the PrimeScript 1st Strand cDNA Synthesis Kit (TaKaRa).

### PCR

PCR was performed using premix Taq (TaKaRa) and specific primers. Primer sequences used to amplify UCP2 were designed based on GenBank sequences: sense primer sequence, 5′-GCCCGGGCTGGTGGTGGTC-3′ and antisense primer sequence, 5′-CCCCGAAGGCAGAAGTGAAGTGG-3′. PCR UCP2 amplification consisted of denaturation at 94°C for 2 min, followed by 25 cycles of 30 s at 95°C, 30 s at 58°C, and 30 s at 72°C, with a final extension for 7 min at 72°C. The PCR products were analyzed using 2% agarose gel electrophoresis, and the UCP2 PCR product size was 290 bp. Glyceraldehyde-3-phosphate dehydrogenase (GAPDH) was used as the control housekeeping gene. GAPDH amplification consisted of denaturation at 94°C for 30 s, followed by 30 cycles of 30 s at 94°C, 30 s at 50°C, and 30 s at 72°C, with a final extension for 10 min at 72°C. The GAPDH sense primer sequence was 5′-ACCACAGTCCATGCCATCAC-3′ and the antisense primer sequence was 5′-TCCACCACCCTGCTGTA-3′. The PCR products were analyzed using 2% agarose gel electrophoresis, and the GAPDH PCR product size was 450 bp.

### Protein extraction and Western blot analysis

The 5-aza-treated cells were homogenized using a sonicator with lysis buffer containing protease inhibitors. Lysates were centrifuged at 12,000 rpm for 20 min at 4°C. Then, the protein lysates were transferred to a new tube. Total protein concentration was assessed using the BCA Protein Assay Kit (Thermo Fisher Scientific, Rockford, IL, USA). Protein samples were boiled at 95°C for 5 min after adding 5× sodium dodecyl sulfate (SDS) polyacrylamide gel electrophoresis loading buffer (25 mM Tris-HCL pH 6.8, 10% SDS, 50% glycerol, 0.5 M dithiothreitol, 0.5% bromophenol blue). Each sample was electrophoresed at a concentration of 500 μg/μl by 10 μl.

After electrophoresis, the proteins were transferred to a nitrocellulose membrane and blocked with 3% skim milk (Sigma-Aldrich, St. Louis, MO, USA) at room temperature for 2 h. Membranes were washed with 1× TBS-T and incubated overnight at 4°C with specific antibodies against UCP2 (1:1,000; Cell Signaling Technology, Danvers, Massachusetts, USA), DNMT1 (1:1,000; Cell Signaling Technology), DNMT3a (1:1,000; Cell Signaling Technology), DNMT3b (1:1,000; Cell Signaling Technology), and β-actin (1:5,000; Cell Signaling Technology). The membranes were washed with 1× TBS-T and incubated for 2 h with horseradish peroxidase (HRP)-conjugated goat anti-rabbit IgG polyclonal antibody (Bethyl, Montgomery, AL, USA) or HRP-conjugated anti-mouse IgG polyclonal antibody (Bethyl Laboratories, Montgomery, TX, USA) at room temperature. Bands were detected using West Pico PLUS Chemiluminescent Substrate (Thermo Fisher Scientific). The visualized bands were shown as a bar graphs through CS Analyzer4 (ATTO Corporation, Tokyo, Japan) that quantifies them in proportion to their thickness and size.

### Measurement of DNMT activity

Nuclear proteins were isolated using the EpiQuik™ Nuclear Extraction Kit I (Epigentek, Brooklyn, NY, USA) from 5-aza-treated cells. After measuring the protein concentration with the BCA Protein Assay Kit (Thermo Fisher Scientific), total DNMT activity was analyzed using EpiQuik™ DNA Methyltransferase Activity/Inhibition Assay (Epigentek). The horizontal dotted line indicate baseline DNMT enzymatic activity of the untreated control group, and the bar graph of the treatment group indicates a value proportional to the control group.

### gDNA purification and bisulfite modification

Genomic DNA (gDNA) was isolated from 5-aza-treated cells using the Wizard® Genomic DNA Purification Kit (Promega Corp., Madison, WI, USA) according to the manufacturer's instructions. Extracted gDNA was modified using the EpiTect® Bisulfite Kit (QIAGEN, Hilden, Germany) according to the manufacturer's instructions. PCR amplification after Bisulfite treatment of DNA chemically converts unmethylated cytosines to thymines without affecting adenines, guanines, thymines or methylated cytosines. This cytosine to thymine conversion results in non-complementary in both strands of DNA. During the experiment, DNA concentration was measured using a NanoDrop (Molecular Devices). Sodium bisulfite-modified DNA was stored at -15 to -30°C.

### MSP and direct PCR sequencing assays

Specific methylated and unmethylated primers are required for MSP analysis. The primer design for non-sulfurized processing sequences is available on the MetPrimer site (http://www.urogene.org/methprimer2/). Specific PCR conditions such as specific methylated and unmethylated primer sequences, combined temperature, and number of cycles of target genes are presented in Table 1. Sodium bisulfite-modified DNA was analyzed using the EpiScope® MSP Kit (TaKaRa) according to the manufacturer's instructions. The PCR products were analyzed using 3% agarose gel electrophoresis. MSP samples were sent to Macrogen Corporation (Seoul, South Korea) and analyzed to examine the methylated sequence in the UCP2 promoter region.

### Statistical analysis

All experiments were repeated five to eight times in biological replicates. The data were expressed as the means ± standard error of the mean. All statistical analyses were performed using analysis of variance with the Statistical Analysis System (SAS) software (SAS Institute, Cary, NC, USA); each treatment was compared using the least-squares or Duncan method. A p-value < 0.05 indicated significant differences among treatments.

## Results

### UCP2 mRNA expression and basal promoter activity of human UCP2 in cancer cells

**Figure 1:**
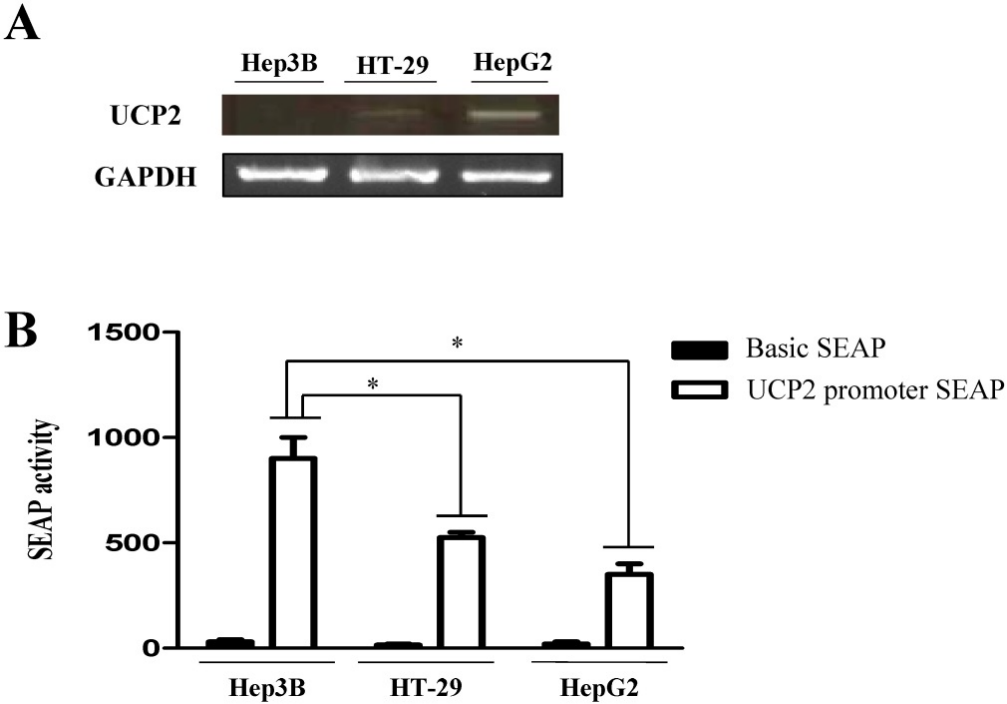
Expression level of human UCP2 mRNA and basal promoter activity of the human UCP2 gene. **(A)** RT-PCR was used to detect the level of UCP2 mRNA expression. **(B)** The UCP2 promoter-SEAP reporter plasmids were transfected into Hep3B, HT-29 and HepG2 cells. The data are expressed as the mean±SEM of five independent transfection experiments. *P < 0.05 vs UCP2 promoter SEAP activity in Hep3B cells.

UCP2 mRNA is not transcribed in Hep3B cells but is transcribed in HT-29 and HepG2 cells 11, 22. In the present study, the transcriptional difference of UCP2 mRNA among the three cancer cell lines was identified (Figure 1A). To determine the difference in the transcription degree of UCP2 mRNA in these cells, total RNA was isolated without any treatment and reverse transcription PCR was performed. UCP2 mRNA was not expressed in Hep3B cells, but was expressed in HT-29 and HepG2 cells, and the highest in HepG2. This finding is in line with earlier studies 11, 21.

A SEAP reporter plasmid containing the UCP2 promoter region was constructed, transfected into each cell line, and basal promoter activity was measured. The SEAP activity in the Hep3B cells in which UCP2 mRNA was not expressed was 900 ± 100 SEAP activity. Conversely, SEAP activities in HT-29 and HepG2 cells in which UCP2 mRNA was expressed were 525 ± 25 and 350 ± 50 SEAP activity, respectively (Figure 1B). The basal activity of the human UCP2 promoter was approximately two-fold higher in Hep3B cells than in HT-29 and HepG2 cells, indicating that transcription of UCP2 mRNA in Hep3B cells was inhibited by some other factors. The HT-29 cells showed slightly higher, albeit nonsignificant, promoter activity than HepG2 cells. In each cell, the average SAEP activity of Basic SEAP reporter plasmid was around 10.

### Effects of 5-aza on UCP2, DNMT level, and DNMT activity in cancer cells

**Figure 2:**
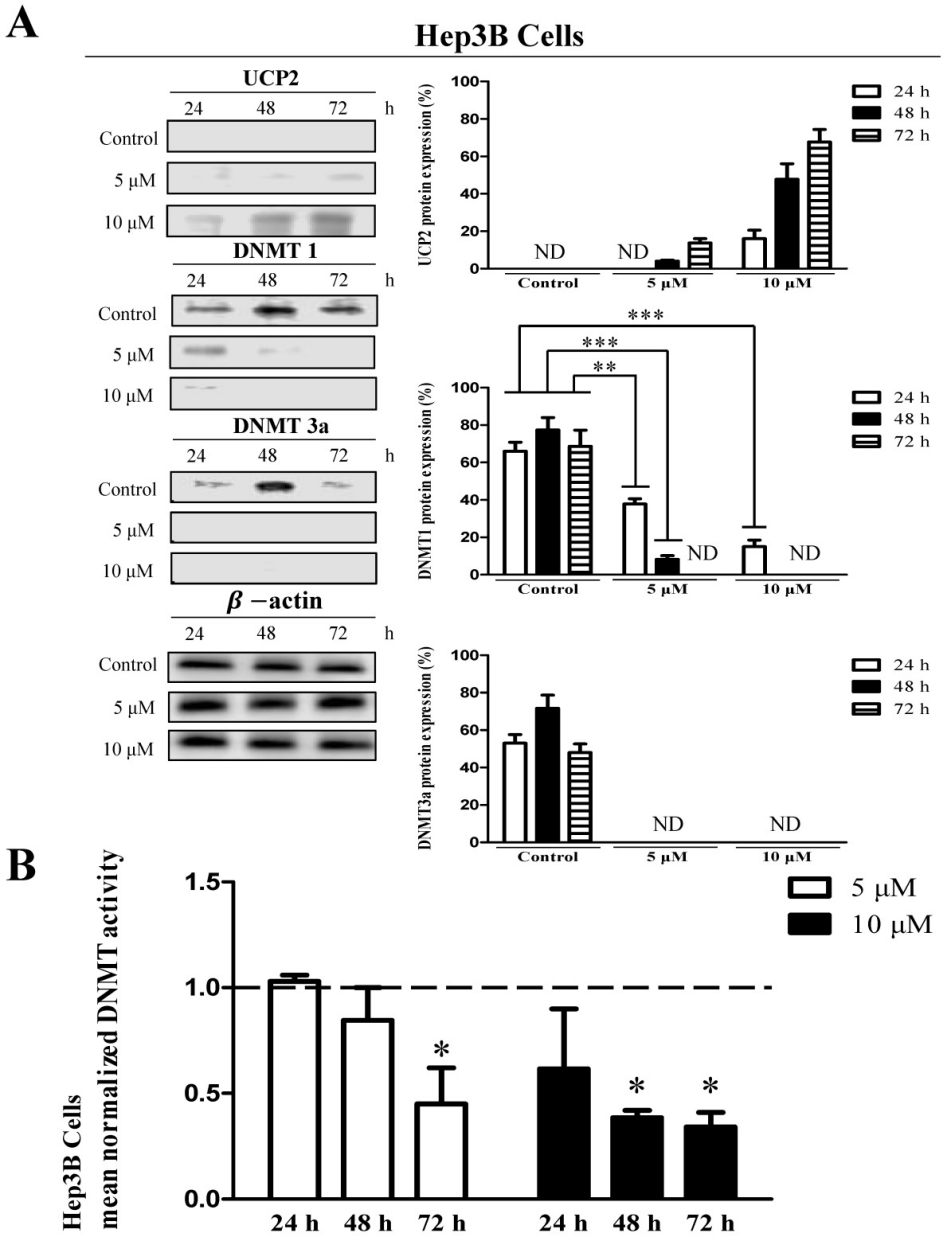
Effects 5-aza on UCP2, DNMT expression level and DNMT enzymatic activity in Hep3B cells. Hep3B cells were cultured for 24, 48, 72 h with 5-aza (0, 5, 10 μM). **(A)** The expression level of UCP2, DNMT1 and DNMT3a were analyzed by Western blot. The data are expressed as the mean±SEM of five independent experiments. **P < 0.01, ***P < 0.001 vs control group. **(B)** Nuclear proteins of Hep3B cells were isolated. The total nuclear protein concentration was normalized and DNMT activity assessed. The horizontal dotted line indicate baseline DNMT enzymatic activity of untreated control Hep3B cells group. The data are expressed as the mean±SEM of five independent experiments. *P < 0.05 vs control group.

To investigate whether methylation in the UCP2 promoter affects UCP2 transcription, cells were treated with 5 or 10 μM 5-aza (a DNA demethylation agent) for 24, 48, or 72 h. In Hep3B cells, UCP2 was not expressed in the control group; however, UCP2 expression increased depending on the concentration and treatment time in the 5-aza-treated groups. In addition, UCP2 expression was highest in cells stimulated for 72 h with 10 μM 5-aza (Figure 2A). DNMT is an enzyme that methylates DNA and affects gene expression 6, 23. In the present study, DNMT level and activity were measured to investigate whether the expression of UCP2 and DNMT are correlated. DNMT1 and DNMT3a expression decreased depending on the concentration and time of 5-aza treatment (Figure 2A). Moreover, DNMT1 and DNMT3a expression were not observed in Hep3B cells treated with 10 μM 5-aza for 48 h (Figure 2A). Whereas DNMT3b expression was not significantly changed by 5-aza (data not shown). The DNMT activity in Hep3B cells was decreased depending on the concentration and treatment time in the 5-aza-treated groups, and the DNMT activity in cells treated with 10 μM 5-aza for 72 h was reduced approximately 2-fold compared with the control group (Figure 2B).

**Figure 3:**
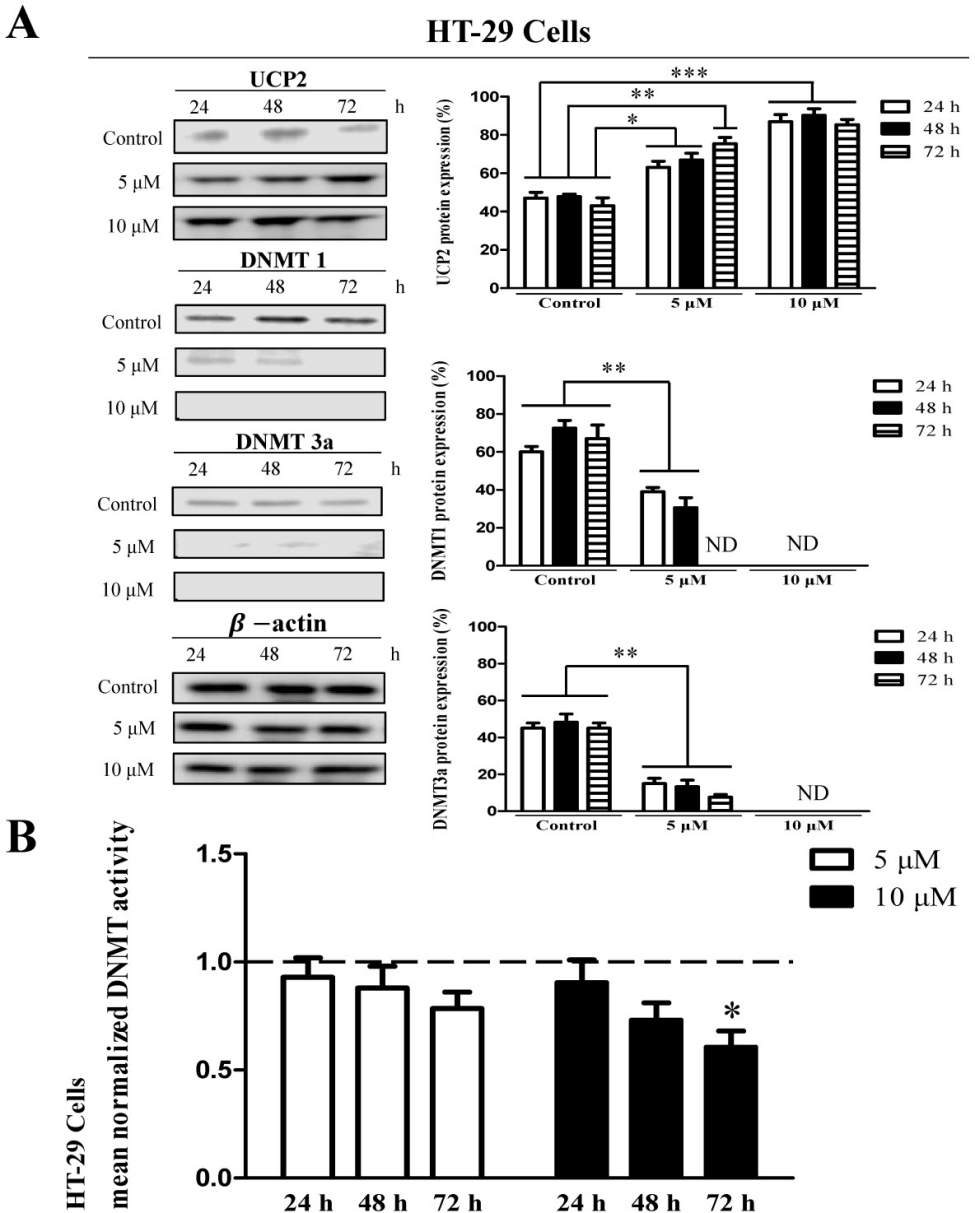
Effects 5-aza on UCP2, DNMT expression level and DNMT enzymatic activity in HT-29 cells. HT-29 cells were cultured for 24, 48, 72 h with 5-aza (0, 5, 10 μM). **(A)** The expression level of UCP2, DNMT1 and DNMT3a were analyzed by Western blot. The data are expressed as the mean±SEM of five independent experiments. *P < 0.05, **P< 0.01, ***P < 0.001 vs control group. **(B)** Nuclear proteins of HT-29 cells were isolated. The total nuclear protein concentration was normalized and DNMT activity assessed. The horizontal dotted line indicate baseline DNMT enzymatic activity of untreated control HT-29 cells group. The data are expressed as the mean±SEM of five independent experiments. *P < 0.05 vs control group.

The results showed that UCP2 was produced in the control group of HT-29 cells (Figure 3A). The UCP2 expression in HT-29 cells was significantly increased depending on the 5-aza concentration and treatment time (Figure 3A). DNMT1 and DNMT3a expression were significantly reduced depending on the 5-aza concentration and treatment time and were not detected in cells treated with 10 μM 5-aza (Figure 3A). However, DNMT3b expression was not significantly changed by 5-aza (data not shown). DNMT activity was significantly decreased in HT-29 cells treated with 10 μM 5-aza for 72 h (Figure 3B).

**Figure 4:**
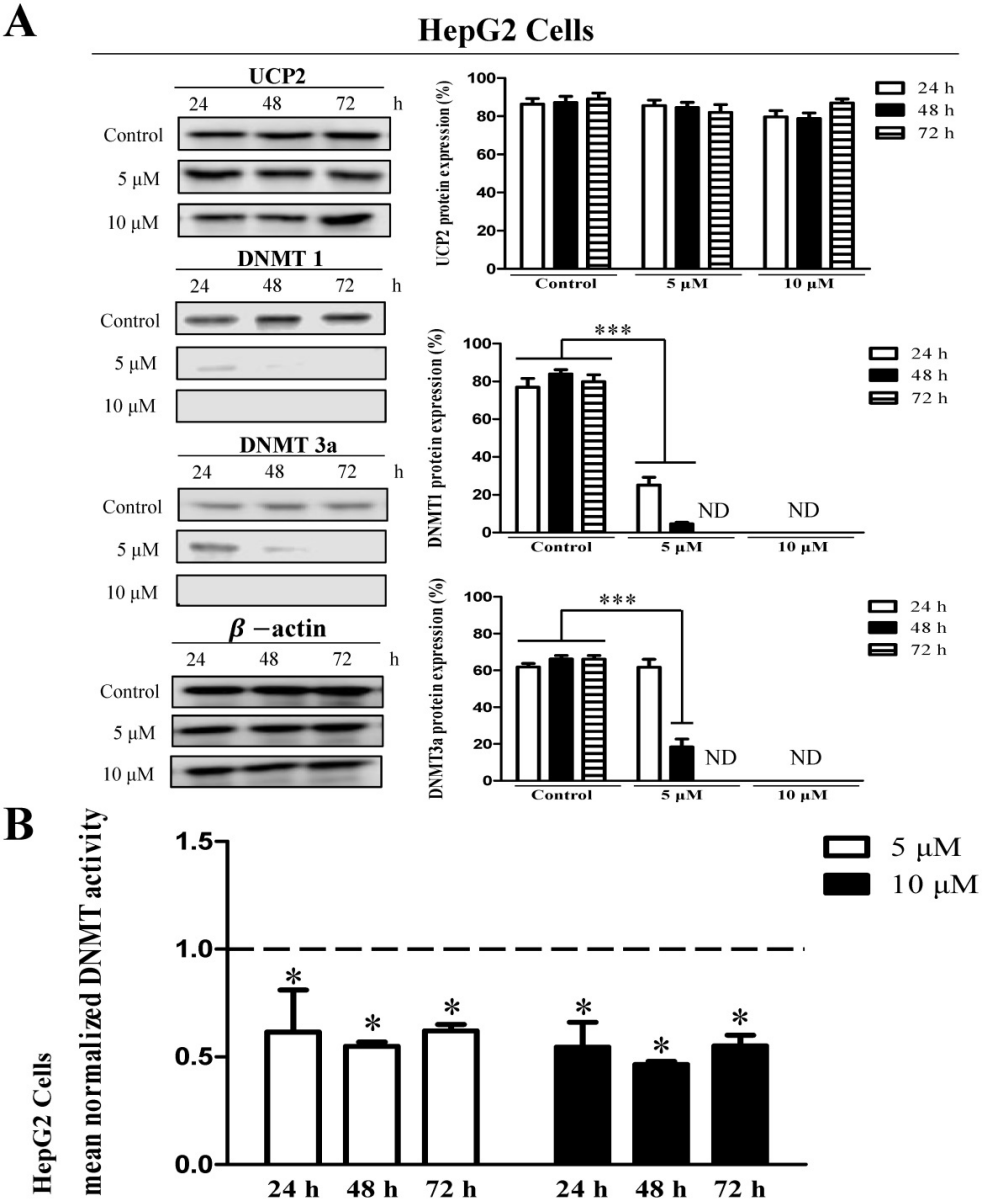
Effects 5-aza on UCP2, DNMT expression level and DNMT enzymatic activity in HepG2 cells. HepG2 cells were cultured for 24, 48, 72 h with 5-aza (0, 5, 10 μM). **(A)** The expression level of UCP2, DNMT1 and DNMT3a were analyzed by Western blot. The data are expressed as the mean±SEM of five independent experiments. ***P < 0.001 vs control group. **(B)** Nuclear proteins of HepG2 cells were isolated. The total nuclear protein concentration was normalized and DNMT activity assessed. The horizontal dotted line indicate baseline DNMT enzymatic activity of untreated control HepG2 cells group. The data are expressed as the mean±SEM of five independent experiments. *P < 0.05 vs control group.

HepG2 cells showed no significant difference in UCP2 expression level in the treatment group compared with the control group (Figure 4A). However, DNMT1 and DNMT3a expression were significantly reduced depending on the 5-aza concentration and treatment time and were not detected in cells treated with 10 μM 5-aza (Figure 4A). However, DNMT3b expression was not significantly changed by 5-aza (data not shown). DNMT activity was also reduced approximately 2-fold in the 5-aza treatment groups compared with the control group (Figure 4A-B).

### Analysis of methylation level in the UCP2 promoter region using MSP

To investigate the level of methylation in the region from -842 to -696 bp in the UCP2 promoter, a specific unmethylated primer confirming the unmethylation degree and a specific methylated primer confirming the methylation degree were created 24. Among the UCP2 promoters, the -842 to -696 bp region was selected as the most appropriate according to the MetPrimer manual. Table 1 shows the primer sequences and PCR conditions. A visible PCR product in lane-U indicates the presence of unmethylated gene promoters; the presence of product in lane-M indicates the presence of promoter methylation.

**Figure 5:**
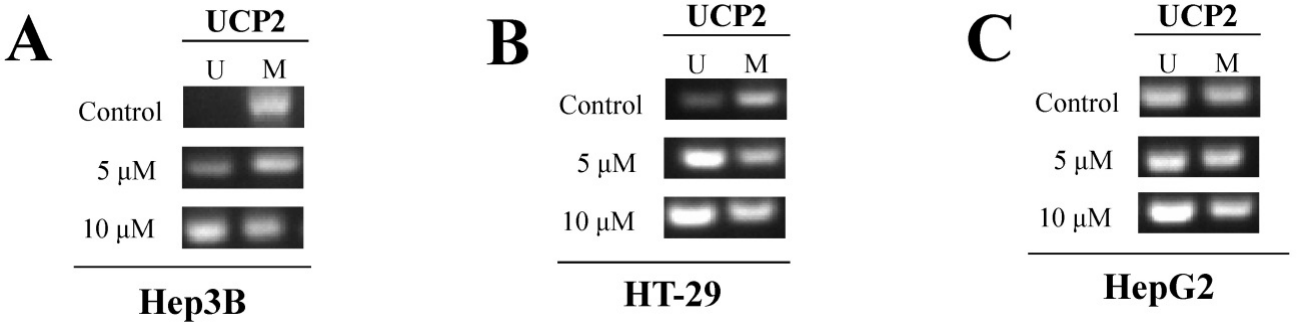
Methylation degree of UCP2 promoter region. Cells were cultured for 24 h with 5-aza (0, 5, 10 μM). Unmethylated DNA was identified using a specific unmethylated primers, and methylated DNA was identified using a specific methylated primers in **(A)** Hep3B cells, **(B)** HT-29 cells, and **(C)** HepG2 cells. U=unmethylated. M=methylated.

Because a specific unmethylated primer was used in the control group of Hep3B cells, gDNA was not amplified in PCR (Figure 5A). However, the degree of gDNA amplification was increased depending on the 5-aza concentration. UCP2 gDNA was amplified when a specific methylated primer was used in the control group of Hep3B cells. These results indicated the methyl group gradually disappeared due to 5-aza treatment and numerous methyl groups existed in the -842 to -696 bp region in the UCP2 promoter of the control group in Hep3B cells. Due to use of a specific unmethylated primer in the control group of HT-29 cells, gDNA was amplified and the increase was 5-aza concentration dependent (Figure 5B). UCP2 gDNA was amplified when a specific methylated primer was used in the control group of HT-29 cells. No significant difference was observed in the gDNA amplification results in the control group compared with the 5-aza treatment groups in HepG2 cells (Figure 5C). It was therefore confirmed that the methyl group is bound through direct PCR sequencing by using MSP sample.

### Analysis of the methylated sequence in the UCP2 promoter region using direct PCR sequencing

**Figure 6:**
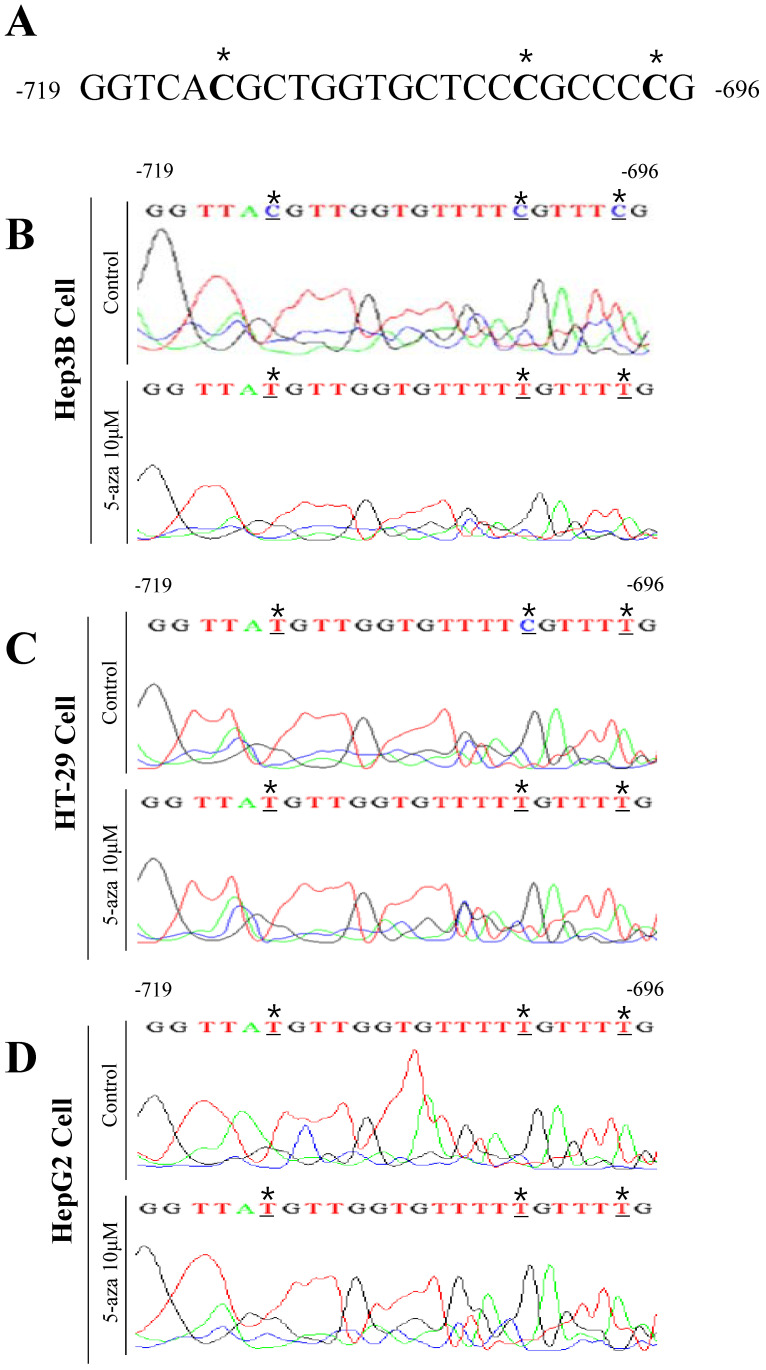
Analysis of methylated sequence in UCP2 promoter by bisulfite sequencing. **(A)** Untreated sodium bisulfite in UCP2 promoter -719 bp to -696 bp region. **(B)** Hep3B cells, **(C)** HT-29 cells, and **(D)** HepG2 cells were cultured for 24 h with 5-aza 10 μM. The bisulfite sequencing analysis was performed in UCP2 promoter from -719 bp to -696 bp regions of (B) Hep3B cells, (C) HT-29 cells and (D) HepG2 cells.

Nucleotide sequence analysis of Hep3B cells showed that the -714, -702, and -697 bps were cytosine because they were not converted into uracil due to the presence of methyl groups (Figure 6B). However, in Hep3B cells treated with 10 μM 5-aza, the -714, -702, and -697 bps in the UCP2 promoter were all thymine (Figure 6B). Subsequent nucleotide sequence analysis of HT-29 cells indicated the -702 bp was cytosine (Figure 6C). While in HT-29 cells treated with 10 μM 5-aza, the -714, -702, and -697 bps in the UCP2 promoter were all thymine (Figure 6C). Sequencing analysis results of HepG2 cells confirmed that the -714, -702, and -697 bps were all thymine (Figure 6D). Whereas in HepG2 cells treated with 10 μM 5-aza, the -714, -702, and -697 bps in the UCP2 promoter were all thymine (Figure 6 D). These results indicated a methyl group was bound to a total of three cytosines at -714, -702, and -697 bp in the UCP2 promoter region from -842 to -696 bp in Hep3B cells. In HT-29 cells, only the cytosine at -702 bp was bound to a methyl group. However, in HepG2 cells, the methyl group was not bound to cytosines at -714, -702, or -697 bp. The results in Figure 6 reveal that the -842 to -696 bp region in the UCP2 promoter of Hep3B cells is methylated and the degree of methylation in HT-29 cells appears to be lower than that in Hep3B cells. In addition, the degree of methylation in HepG2 cells was lower than that in HT-29 cells.

## Discussion

UCP2 expression is inhibited in Hep3B cells 11. However, UCP2 expression normally occurs in HepG2 and HT-29 cells 11, 22. In the present study, the UCP2 mRNA transcription and protein expression levels differed among the cell lines. Various factors can affect UCP2 expression in cells; nonetheless, the activity of transcriptional stimulating factors is considered important. In this study, a plasmid containing the promoter region of UCP2 was constructed, transfected into three cancer cell lines, and the activity of the UCP2 promoter was measured. The basal promoter activity in Hep3B cells in which UCP2 was not produced was significantly higher compared with HT-29 and HepG2 cells in which UCP2 was normally produced. These results indicate that transcription factors involved in UCP2 expression are functioning normally in Hep3B, HT-29, and HepG2 cells. However, because UCP2 expression was suppressed only in Hep3B cells, another factor likely interferes with transcription in the UCP2 promoter in these cells.

DNA methylation, an epigenetic mechanism, does not alter the DNA sequence but usually attaches the methyl group to the cytosine base of CpG 5. Methylation in the promoter region of a specific gene prevents transcription by interfering with the binding of transcription stimulating factors 4, 9, 10. DNMT is an enzyme that regulates DNA methylation 6 and catalyzes the transfer of methyl groups to cytosine in the CpG sequence 25. In mammals, there are three DNMTs, DNMT1, DNMT3a, and DNMT3b 8. DNMT1 (maintenance DNMT) is an enzyme that methylates the CpG region synthesized during the cell differentiation process 26, 27. DNMT3a and DNMT3b (*de novo* DNMTs) are enzymes that methylate DNA CpG dinucleotides in early cells before cellular differentiation 28, 29. Intracellular methylation can also be affected by a decrease in the activity of one of the three DNMTs 28, 29. Many studies have investigated DNMT function. Reportedly, DNMT1 can function as DNMT3a and DNMT3b, and DNMT3a and DNMT3b can also act as DNMT1 30, 31. The transcription and protein expression of DNMT increases during cell proliferation and is more pronounced in tumor cells than in normal cells. In addition, the DNMT expression level in cancer cells is high 32. DNMT affects the methylation of several genes, including p53, a tumor suppressor gene 33-36. p53 protein is not expressed in Hep3B cells; however, inhibition of DNMT expression induces p53 protein expression 37. DNA demethylation caused by 5-aza activates the p53 signaling pathway and induces apoptosis 35. Furthermore, inhibition of DNMT activity leads to demethylation of the p53 promoter, resulting in apoptosis 38. In addition, DNA demethylation of p15 and p16 genes can inhibit HCC proliferation 6, 39. In our study, when cells that do not express UCP2 were treated with 5-aza, a DNA demethylation drug 22, 23, DNMT activity was decreased, DNA methylation was inhibited, and UCP2 expression was increased. Combining these facts with the results of previous studies, it is thought that 5-aza inhibits DNMT activity, thereby increasing the expression of UCP2. The results in Figures 2B, 3B, and 4B showed that treatment with 5-aza in cells did not completely reduce DNMT activity. This is thought to be because DNMT3b expression was not affected by 5-aza.

Based on the results presented in Figure 1, we hypothesized that UCP2 expression in Hep3B cells is inhibited by DNA methylation, and that the UCP2 expression level in HT-29 and HepG2 cells may differ depending on the degree of methylation. Therefore, each cell line was treated with the DNMT inhibitor 5-aza 22, 23 to observe UCP2 expression level and methylation changes in the UCP2 promoter region. In Hep3B and HT-29 cells, UCP2 expression was increased depending on the concentration of 5-aza; however, DNMT expression and activity were decreased (Figure 2 and 3). These results indicate that UCP2 and DNMT expression levels in Hep3B and HT-29 cells are inversely correlated. On the other hand, treatment with 5-aza significantly decreased DNMT expression and activity in HepG2 cells but did not affect UCP2 expression (Figure 4), indicating that UCP2 and DNMT expression levels are not correlated in HepG2 cells.

The MSP test results showed that UCP2 gDNA in the control group of Hep3B cells was not amplified due to a methyl group at the UCP2 promoter in the -842 to -696 bp region; however, treatment with 5-aza significantly amplified UCP2 gDNA (Figure 5A). In the control group of HT-29 cells, UCP2 gDNA was amplified in the UCP2 promoter in the -842 to -696 bp region, and 5-aza treatment further amplified gDNA (Figure 5B). In the control group of HepG2 cells, UCP2 gDNA was amplified in the UCP2 promoter in the -842 to -696 bp region, and treatment with 5-aza did not significantly amplify gDNA (Figure 5C). The results indicate that the UCP2 promoter region in Hep3B cells undergoes significant methylation compared with HT-29 cells, and the UCP2 promoter region in HT-29 cells is more methylated than in Hep2G cells. Future studies are necessary to investigate the degree of methylation for the entire UCP2 promoter region; however, the methylation of the sequences from -842 to -696 bp in the UCP2 promoter region in Hep3B cells likely inhibits UCP2 expression. In Figure 5A, B, and C there is not a complete reduction of methylated UCP2 after treatment with 5-aza. It is due to the absence of DNMT3b expression modulation after the treatment with 5-aza.

In the present study, MSP samples were analyzed to examine the methylated sequence. When gDNA is treated with sodium bisulfite, the unmethylated cytosine sequence is transfected into uracil. At the time of performing PCR with this gDNA, the uracil sequence is converted into thymine. The control group of Hep3B cells was treated with sodium bisulfite and methyl groups were observed in cytosines at -714, -702, and -697 bp (Figure 6B). However, in HT-29 cells, the methyl group was only observed in the cytosine at -702 bp, and in HepG2 cells, the methyl group was not present (Figure 6C-D). In future studies, methylation in the entire promoter sequence should be analyzed to confirm that the nucleotide sequences in the UCP2 promoter region from -842 to -696 bp are important sites for transcription stimulatory factors. The results of the present study showed that the UCP2 promoter region in Hep3B cells has numerous methylated sites compared with other cancer cells and that UCP2 expression is inhibited by methylation. Furthermore, treatment with 5-aza increased UCP2 level in Hep3B and HT-29 cells but not in HepG2 cells.

In cancer cells, UCP2 may play a role in causing apoptosis 15, 16, while several UCP2 functions remain unclear. Conversely, UCP2 has been shown to help tumor cells proliferate by inhibiting ROS production 13, 14 and promote pancreatic cancer proliferation 40. In the future, the research on function of UCP2 related to apoptosis in the cancer cell is in need. However, the results of this study proved that when 5-aza was treated with Hep3B and HT-29 cells, the methylation of the promoter of UCP2 was decreased and the expression of UCP2 was increased. This result provides an important opportunity to study the function of UCP2 related to cancer treatment.

## Conclusions

The UCP2 promoter in Hep3B cells is methylated and the degree of UCP2 promoter methylation in HT-29 cells is lower than that in Hep3B cells. In addition, the degree of methylation in HepG2 cells is lower than that in HT-29 cells. Therefore, 5-aza treatment can increase the UCP2 expression level in Hep3B and HT-29 cells but not in HepG2 cells.

## Figures and Tables

**Table 1:** Primer sequences and PCR conditions for MSP analysis

Gene Name		Primer sequence (5'-3')		Product size (bp)	Annealing temperature
		Forward	Reverse		
UCP2	U	GGATGGGTTAGTTAATTAAAGGT	ACCAAAACACTAAAAACCCCAA	188	55℃
	M	ACGGGTTAGTTAGTTAATTAAAGGC	CCAAAACACTAAAAACCCCG	191	55℃

PCR was carried out in 43 cylces. (U) a specific unmethylated primer sequence, (M) a specific methylated primer sequence.

## Abbreviations

5-aza: 5-azacytidine
cDNA: complementary DNA
DMEM: Dulbecco's minimum Eagle's medium
DNMT: DNA methyltransferase
FCS: fetal calf serum
GAPDH: glyceraldehyde-3-phosphate dehydrogenase
gDNA: genomic DNA
HCC: hepatocellular carcinoma
HRP: horseradish peroxidase
MSP: methylation-specific PCR
SDS: sodium dodecyl sulfate
UCP2: Uncoupling protein 2
